#  Myoferlin at the crossroad of vesicle trafficking and mitochondrial function: implications for pancreatic cancer progression and stromal reprogramming

**DOI:** 10.1042/BST20260427

**Published:** 2026-07-30

**Authors:** Raphaël Peiffer, Emilie Laverdeur, Aline Genbauffe, Akeila Bellahcène, Gilles Rademaker, Olivier Peulen

**Affiliations:** 1 Metastasis Research Laboratory, GIGA-Institute, University of Liège, Liège, Belgium; 2 Personalized Oncology Division, The Walter and Eliza Hall Institute of Medical Research, Parkville, VIC, Australia

**Keywords:** cancer-associated fibroblasts, COPII vesicles, mitochondria, myoferlin, RAB proteins

## Abstract

Myoferlin, a type 2 transmembrane protein in the ferlin family, is traditionally known for its role in membrane fusion during muscle development and repair. Recent research identifies myoferlin as a potential biomarker and a critical driver of cancer progression, particularly in breast cancer and pancreatic ductal adenocarcinoma. While its lack of specificity limits its use as a biomarker, its multifaceted role in cellular membrane dynamics makes it a promising therapeutic target. In cancer cells, myoferlin regulates the recycling and stability of receptor tyrosine kinases, thereby promoting invasion and metastasis. Beyond the plasma membrane, it maintains mitochondrial homeostasis by interacting with the machinery for mitochondrial fusion and calcium exchange at the endoplasmic reticulum–mitochondria interface. Depletion of myoferlin disrupts these processes, leading to mitochondrial fragmentation, reduced ATP production, and iron-dependent cell death. Furthermore, myoferlin influences the tumour microenvironment by regulating pancreatic cancer-associated fibroblasts. It interacts with SEC24 to facilitate the coat protein complex II-mediated transport of the transforming growth factor-beta 1 receptor, driving the desmoplastic reaction and matrix protein deposition. The ‘one punch–two hits’ strategy—simultaneously targeting the metabolic and signalling pathways of both malignant cells and the stroma—offers a novel therapeutic perspective. The development of small molecules targeting myoferlin’s C2 domains confirms its potential to reduce tumour growth and metastatic dissemination.

## Introduction

The ferlin family is an ancient gene family common to eukaryotes, ranging from unicellular phytoplankton and apicomplexans to metazoans, but absent in plants and fungi [1]. Most vertebrates possess six ferlin family genes (*DYSF, OTOF, MYOF, FER1L4, FER1L5*, and *FER1L6* in humans), which emerged from an ancestral gene between the divergence of agnathans and cartilaginous fish during genome duplications. Myoferlin, encoded by the *MYOF* gene, is the most investigated in cancer. Myoferlin is a type 2 transmembrane protein characterised by the presence of multiple C2 domains, which allow it to bind to biological membrane phospholipids in response to variations in calcium concentration [2,3].

## Myoferlin function in normal cells

Like its orthologue *fer-1* in the nematode *Caenorhabditis elegans*, myoferlin plays an important role in membrane fusion. Indeed, during development, myoferlin is involved in muscle development by enabling the fusion of precursor cells (myoblasts) into muscle fibres (myotubes) [4]. In adulthood, it participates in muscle repair by promoting the fusion of intracellular vesicles with damaged regions of the plasma membrane [4]. However, interrogation of ‘The Human Protein Atlas’ (https://www.proteinatlas.org) shows that its expression is not limited to muscle cells, but extended to endocrine tissues, respiratory system, liver, kidney, and urinary bladder, suggesting other functions.

In non-pathological conditions, and beyond the muscular system, myoferlin function has primarily been investigated in two cell types: phagocytes and endothelial cells. Within phagocytes, myoferlin is described as a regulator of calcium-triggered lysosomal exocytosis [5]. Meanwhile, in endothelial cells, its presence is necessary for receptor-mediated endocytosis and the transport of receptor tyrosine kinases to the plasma membrane: the VEGFA receptors (VEGFR2) and angiopoietin receptors (TIE2) [6–8]. A similar role has also been attributed to myoferlin in muscle cells regarding another receptor tyrosine kinase, the insulin-like growth factor receptor (IGF1R) [9]. In each case, myoferlin’s function remains clearly associated with the fusion of cellular membranes. Its interaction with EHD2 and RAB family GTPases (RAB7 and RAB5) could be at the heart of this mechanism [10,11].

## From biomarker to therapeutic target

In the context of oncology, myoferlin was initially identified as a potential biomarker due to its overexpression in malignant tissues compared with non-pathological counterparts. Early global gene expression [12–14] and proteomic [15–17] studies highlighted myoferlin as a differentially expressed protein in cancers. However, while its dramatic up-regulation initially pointed towards a diagnostic or prognostic biomarker role, its lack of tissue and cancer specificity subsequently shifted research interest from a mere detection marker towards functional investigations and its evaluation as an innovative therapeutic target, mainly in breast and pancreatic cancers.

### Breast cancer cells

**Figure 1:**
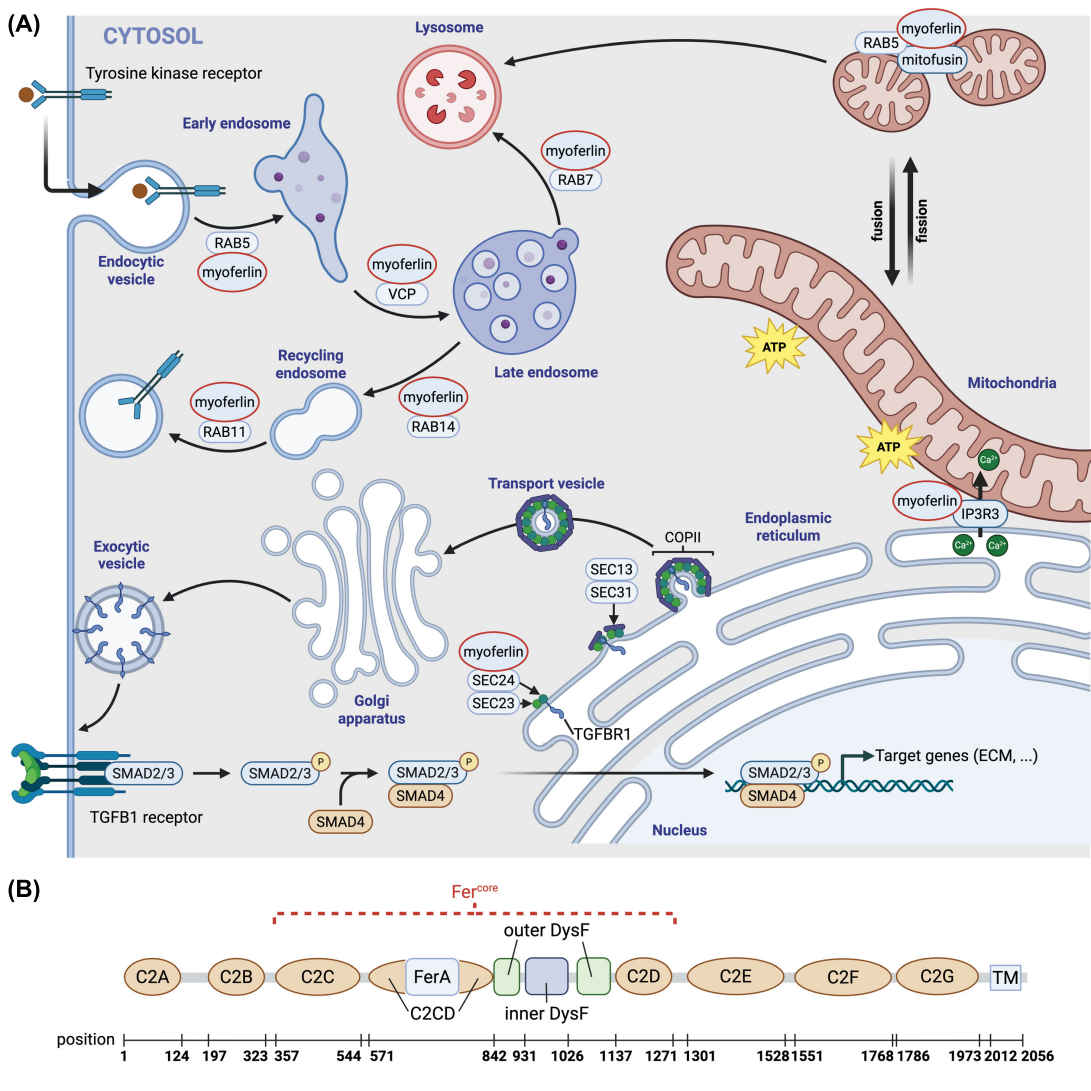
Pivotal role of myoferlin in the cellular protein interactome and topological representation of myoferlin (**A**) Summary of physical interactions of myoferlin involved in recycling of receptor tyrosine kinases, mitochondrial calcium dynamics and homeostasis (endoplasmic reticulum–mitochondria exchanges), and cargo sorting of coat protein complex II (COPII) vesicles. COPII: coat protein complex II, ECM: extracellular matrix. (**B**) Topological representation of myoferlin. Adapted from [50] according to http://creativecommons.org/licenses/by/4.0/. Created in BioRender. Peulen, O. (2026) https://BioRender.com/ey66zwy

The first study to explore myoferlin’s function beyond its mere detection in cancer cells or tissues dates back to 2011. The authors developed a mathematical model suggesting that myoferlin plays a role in the secretion of matrix metalloproteinases and the recycling of internalised receptors back to the plasma membrane [18]. This model was validated in breast cancer cells, where myoferlin expression was inhibited using RNA interference. These cells showed a reduced invasion capacity in a Matrigel™ matrix, corroborating the hypothesis of decreased matrix metalloproteinase production [18,19]. Furthermore, several receptor tyrosine kinases, including VEGFR2 and IGF1R, exhibited reduced activity, suggesting a possible absence from the plasma membrane (Figure 1A) [18–22]. Additionally, epidermal growth factor receptor (EGFR) recycling was also reported as impaired [23]. These observations consistently involve events where membrane fusions occur, either as exocytosis or as endocytosis and fusion with endosomes. The impact of these modifications appears major, as myoferlin-deficient breast cancer cells produce less aggressive and smaller tumours [20,23–25]. Similar results have been observed in colon and lung tumour models, among others [22,26,27]. Consistent with what is observed in normal cells, these findings may originate from the interaction of myoferlin with RAB5 and RAB7 proteins [20,22]. Additionally, a study in HeLa cells identified myoferlin as an interacting partner of RAB7, RAB11, and RAB14, as well as p97/VCP (valosin-containing protein); notably, its interaction with p97/VCP is mediated by the phospholipase A-2-activating protein [28]. The authors suggest that the myoferlin-p97/VCP complex likely assembles at the recycling endosomal compartment, where both proteins colocalise.

### Pancreatic cancer cells

While the pancreas was the tumour site where myoferlin was first identified outside a muscular context, it is also where its function has been extensively studied over the last 10 years. Two main pathways emerge from the present research, though they may be two manifestations of the same mechanism. The first involves the endomembrane system and cellular vesicular trafficking, while the second concerns mitochondrial dynamics and function.

In line with its canonical membrane function, genetic depletion of myoferlin in pancreatic cells reduces VEGFA exocytosis, while its pharmacological inhibition alters the recycling of VEGFR2 and another receptor tyrosine kinase, EGFR (Figure 1A) [21,29]. The impact of myoferlin depletion on membrane protein recycling extends beyond receptor tyrosine kinases; a recent report demonstrates a profound disruption of focal adhesion point recycling [30]. In direct relation with the endomembrane system and membrane protein recycling, myoferlin was reported as exceptionally abundant at lysosomal membranes in pancreatic cancer, where it is required for the maintenance of lysosome health and the protection against membrane damage [31].

Myoferlin has also been implicated in the biology of exosomes produced by pancreatic cancer cells [32]. In addition to being present in exosomes, inhibiting myoferlin expression via RNA interference reduces the average size of exosomes, profoundly modifies their protein cargo composition, and renders them non-functional, as they are no longer able to deliver their contents to endothelial cells. To date, no mechanism has been proposed to explain these effects. However, it is plausible that the presence of myoferlin in the exosomal membrane participates in membrane fusion with the target cells. Regarding the modulation of the protein cargo within exosomes, more specific investigations evaluating, for example, the potential relationship between myoferlin and endosomal sorting complexes required for transport proteins are necessary.

The mitochondrion is the primary organelle for energy production in the form of ATP in eukaryotic cells. Cancer cells exhibit metabolic hyperactivity; their proliferation requires massive and constant ATP production, placing their mitochondria under intense functional pressure. Although myoferlin is not present within the mitochondrion itself or its membranes, it appears to be near or in interaction with proteins involved in mitochondrial fusion events (mitofusins—MFN1/2) (Figure 1A) [33,34]. The transition from a fragmented state to an interconnected network of mitochondria through fusion acts as a metabolic optimisation mechanism, allowing for greater ATP production by sharing resources and propagating membrane potential. Genetic depletion of myoferlin in malignant pancreatic cells shifts the mitochondrial balance towards the fragmented form of this organelle, via the phosphorylation of dynamin-related protein 1 (DRP1), and leads to a reduction in mitochondrial respiratory activity [35]. Interestingly, observations indicate an interaction between MFN1 and RAB5 [36], as well as between DRP1 and RAB32 at the contact points between the endoplasmic reticulum and the mitochondrion (mitochondrial-associated membranes, MAMs) [37]. These reports raise the possibility that the mitochondrial phenotype induced by myoferlin depletion stems from a disruption of a RAB-dependent pathway.

The origin of this reduced mitochondrial metabolic activity may also lie in the disruption of calcium exchanges between the endoplasmic reticulum and the mitochondrion. Recent analyses demonstrate that myoferlin is localised in the MAMs, near the VDAC1 calcium channel, where it interacts with the type 3 inositol 1,4,5-trisphosphate receptor (IP3R3) (Figure 1A) [34]. Myoferlin depletion could therefore cause destabilisation or improper assembly of the IP3R3–GRP75–VDAC1 supercomplex, explaining the reduction in calcium exchange. These two mechanisms—fission caused by DRP1 phosphorylation and reduced respiratory activity explained by reduced mitochondrial calcium concentration—may be linked. Indeed, changes in mitochondrial shape and membrane curvature may cause misalignment of IP3R3 and VDAC1, explaining the reduced calcium flux to the mitochondrion. Recent studies show that, beyond mitochondrial fission, myoferlin depletion and pharmacological inhibition of myoferlin function drive mitochondria towards mitophagy and result in iron-dependent cell death, ferroptosis [38,39].

### Pancreatic cancer-associated fibroblasts

Reducing pancreatic tumours solely to cancer cells is overly simplistic, as they are among the most desmoplastic tumours. Numerous cancer-associated fibroblasts (CAFs) produce an abundant protein matrix that constitutes a biological and physical barrier restricting access to immune cells and drugs. The observation of myoferlin’s presence in these fibroblasts prompted us to investigate its stromal function [40]. Correlative analyses of patient samples indicate that myoferlin RNA expression and protein abundance are associated with increased matrix proteins such as collagen and fibronectin.

The development of experimental models based on CAFs isolated from pancreatic cancer patients has allowed us to move beyond the correlative aspect of our observation. *MYOF* depletion in these cells reduces both the expression of genes encoding collagen and fibronectin and the deposition of these matrix proteins in three-dimensional models. The expression of these genes is controlled by the TGF-beta pathway starting with the transforming growth factor-beta 1 (TGFB1) receptor, TGFBR1, and leading to the nuclear translocation of a specific transcription factor (SMAD2/3) (Figure 1A) [40].

Given myoferlin’s described functions in the trafficking of receptor tyrosine kinases to the plasma membrane, we considered that the transport of the specific serine/threonine kinase receptor for TGFB1 might also be under myoferlin’s control. *MYOF* depletion reduces the binding of the TGFB1 ligand to the membrane, as well as the activation of the downstream cascade and the subsequent translocation of SMAD2/3 to the nucleus [40]. It is therefore reasonable to think that these results are a consequence of reduced TGFBR1 membrane density.

**Figure 2:**
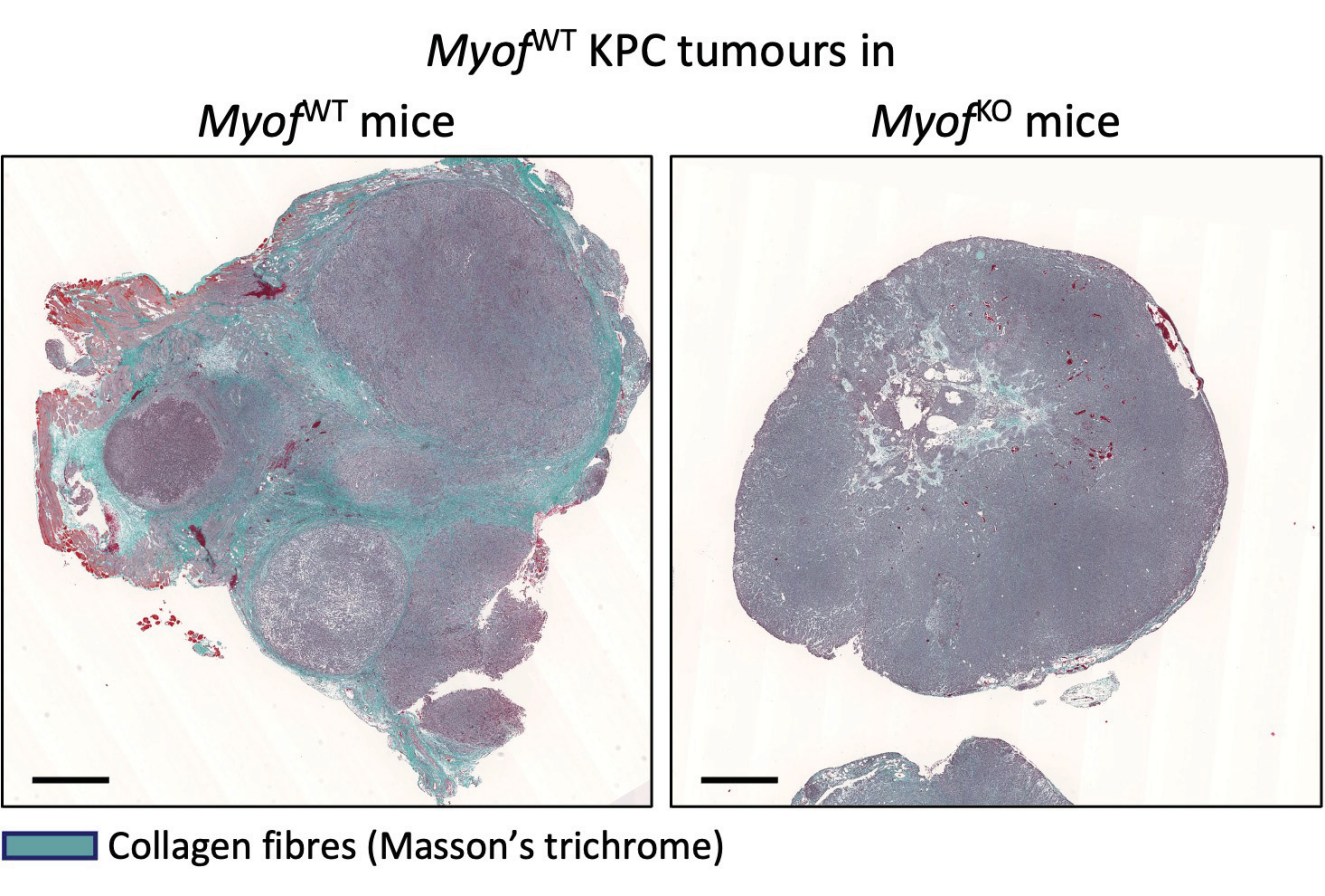
Histochemistry of pancreatic tumours developed in myoferlin-deficient mice Murine pancreatic cancer cells (KPC) were implanted into the pancreas of conventional recipient mice (*Myof*^WT^) or myoferlin-deficient mice (*Myof*^KO^). Tumours were stained with Masson’s trichrome to highlight collagen fibres. Scale bar represents 1 mm. Adapted from [40] according to http://creativecommons.org/licenses/by/4.0/.

Unbiased analysis of RNA sequencing data reveals that a genetic program linked to protein transport between the endoplasmic reticulum and the Golgi apparatus is inhibited by *MYOF* depletion [40]. This transport occurs via vesicles coated with coat protein complex II. These vesicles form at the endoplasmic reticulum exit site (ERES) through the progressive self-assembly of secretion proteins (SEC) [41]. One of the first events in this assembly is the recruitment of the future protein cargo by a dimer of SEC23 and SEC24 proteins [42]. Within this pair, SEC24 identifies and binds to proteins carrying a specific sorting signal, such as the one carried by TGFBR1. This assembly induces and stabilises a curvature of the endoplasmic reticulum membrane. To complete the vesicle, a polyhedral cage made of SEC13 and SEC31 proteins forms the outer coat and forces the endoplasmic reticulum membrane to evaginate further until it separates [43]. Our results indicate that myoferlin interacts specifically with the SEC24 protein specialised in cargo selection (Figure 1A) [40]. In the absence of myoferlin, SEC24-positive vesicles and TGFBR1 are less abundant in the Golgi apparatus, supporting our hypothesis of reduced TGFBR1 membrane density. Very recently, a similar discovery but related to collagen fibres was made in a chondrosarcoma cell line [44]. Consequently, suppressing the *MYOF* gene in the stromal compartment, as well as pharmacological targeting of myoferlin, reduces the desmoplastic reaction within pancreatic tumours (Figure 2), opening new treatment perspectives. In line with the involvement of RAB proteins described above, RAB1 localizes to ERES, where it regulates anterograde vesicle trafficking from the endoplasmic reticulum to the Golgi apparatus [45].

## A ‘one punch–two hits’ strategy

The results described above suggest that myoferlin could be used as a therapeutic target in pancreatic cancer. This hypothesis is supported by the observed correlation between *MYOF* gene expression levels and the survival of pancreatic cancer patients [17,31,35,39,46]. Experimentally, myoferlin's potential as a therapeutic target is confirmed by several publications demonstrating that genetic targeting of myoferlin in cancer cells reduces tumour growth and metastatic dissemination to the liver [31,38,39,47].

This clinical potential is further enhanced by the recent development of original molecules targeting the C2D domain (Figure 1B) of myoferlin [20–22,27,48] and the discovery of an interaction between picroside II and myoferlin [39].

C2 domains, harboured by proteins such as synaptotagmins, are typically responsible for tethering intracellular vesicles to target membranes while promoting membrane fusion. Ferlin proteins, which are predicted to possess up to 8 C2 domains [49], play a paramount role in this process. A recent report based on cryo-EM analysis has provided significant insights into lipid binding of human ferlins [50]. The authors demonstrated that myoferlin engages lipid membranes through its C2B-C2G, C2C, and C2F-C2G domains. They also revealed that the inner-DysF motif forms direct membrane contacts, the adequate positioning of which appears to be influenced by interactions with the C2CD and C2D domains. Additionally, the study suggests that the C2D domain, while situated on the cytosolic side of the protein, promotes the recruitment of the C2F-C2G domains to the membrane. Interestingly, it is precisely the binding of the synthetic compounds to this C2D domain that disrupts the interaction of myoferlin with RAB7/32 proteins [20,22], thereby suggesting an involvement of this domain in the interaction between myoferlin and RAB proteins. However, cryo-EM analysis of myoferlin does not show any binding of these synthetic compounds, as the described binding site is not accessible [50]. Concurrently, molecular docking simulations suggested that picroside II—a glycoside derivative found in traditional Chinese herbal medicine—binds to the C2CD domain of myoferlin, specifically targeting residues Thr765, Glu766, Glu767, Asn770, and Arg790. This interaction subsequently triggers the lysosome-mediated degradation of myoferlin.

Myoferlin-targeting compounds demonstrate effects comparable to those observed upon genetic depletion in cancer cells but also in CAFs. They lead to ferroptotic cancer cell death [38] and desmoplasia reduction [40]. Consequently, targeting myoferlin, ideally pharmacologically, could represent a considerable advantage for the patient by simultaneously targeting cancer cells and CAFs in the stromal compartment. Indeed, we have observed that when considering myoferlin protein abundance rather than gene expression, it is its presence in the stroma that determines patient survival [40]. Myoferlin thus emerges as a unique target (‘one punch’) capable of reaching two essential compartments (‘two hits’) involved in tumour development and aggressiveness.

## Perspectives

- Prognosis for pancreatic cancer remains grim, and surgery is still the only curative therapy; exploring innovative strategies that target mechanisms not specific to cancer cells may offer a vital new path forward.
- The pharmacological targeting of myoferlin could represent an innovative ‘one punch–two hits’ strategy benefiting patients by simultaneously targeting cancer cells and cancer-associated fibroblasts.
- The newly developed compounds targeting myoferlin must now be evaluated from a clinical trial perspective.

## Open Access

Open access for this article is provided via our Subscribe to Open programme, made possible through the support of our subscribing institutions. Learn more here: https://portlandpress.com/pages/open_access_options_and_prices#conditional.

## Abbreviations

CAFs: cancer-associated fibroblasts
COPII: coat protein complex II
DRP1: dynamin-related protein 1
EGFR: epidermal growth factor receptor
ERES: endoplasmic reticulum exit site
ESCRT: endosomal sorting complexes required for transport
IGF1R: insulin-like growth factor receptor
IP3R3: inositol 1,4,5-trisphosphate receptor, type 3
MAMs: mitochondrial-associated membranes
SEC: secretion proteins
TGFB1: transforming growth factor-beta 1
TGFBR1: transforming growth factor-beta receptor 1
